# Perioperative imaging in patients treated with resection of brain metastases: a survey by the European Association of Neuro-Oncology (EANO) Youngsters committee

**DOI:** 10.1186/s12885-020-06897-z

**Published:** 2020-05-12

**Authors:** Barbara Kiesel, Carina M. Thomé, Tobias Weiss, Asgeir S. Jakola, Amélie Darlix, Alessia Pellerino, Julia Furtner, Johannes Kerschbaumer, Christian F. Freyschlag, Wolfgang Wick, Matthias Preusser, Georg Widhalm, Anna S. Berghoff

**Affiliations:** 1 Department of Neurosurgery, Medical University Vienna, Vienna, Austria; 2 Comprehensive Cancer Center, Medical University of Vienna, Vienna, Austria; 3 Clinical Cooperation Unit Neurooncology, German Cancer Consortium (DKTK), German Cancer Research Center (DKFZ), Heidelberg, Germany; 4 Department of Neurology and Brain Tumor Center, University Hospital and University of Zurich, Zurich, Switzerland; 5 Department of Neurosurgery, Sahlgrenska University Hospital, Gothenburg, Sweden; 6 Department of Medical Oncology, Institut Régional Du Cancer Montpellier, University of Montpellier, Montpellier, France; 7 Department of Neuro-Oncology, University and City of Health and Science Hospital of Turin, Turin, Italy; 8 Department of Biomedical Imaging and Image-guided Therapy, Medical University Vienna, Vienna, Austria; 9 Department of Neurosurgery, Medical University Innsbruck, Innsbruck, Austria; 10 Neurology Clinic & National Center for Tumor Disease, University of Heidelberg, Heidelberg, Germany; 11 Department of Medicine I, Clinical Division of Oncology, Medical University of Vienna, Waehringer Guertel 18-20, 1090 Vienna, Austria

**Keywords:** Postoperative MRI, International guidelines, Perioperative imaging, Brain metastases

## Abstract

**Background:**

Neurosurgical resection represents an important treatment option in the modern, multimodal therapy approach of brain metastases (BM). Guidelines for perioperative imaging exist for primary brain tumors to guide postsurgical treatment. Optimal perioperative imaging of BM patients is so far a matter of debate as no structured guidelines exist.

**Methods:**

A comprehensive questionnaire about perioperative imaging was designed by the European Association of Neuro-Oncology (EANO) Youngsters Committee. The survey was distributed to physicians via the EANO network to perform a descriptive overview on the current habits and their variability on perioperative imaging. Chi square test was used for dichotomous variables.

**Results:**

One hundred twenty physicians worldwide responded to the survey. MRI was the preferred preoperative imaging method (93.3%). Overall 106/120 (88.3%) physicians performed postsurgical imaging routinely including MRI alone (62/120 [51.7%]), postoperative CT (29/120 [24.2%]) and MRI + CT (15/120 [12.5%]). No correlation of postsurgical MRI utilization in academic vs. non-academic hospitals (58/89 [65.2%] vs. 19/31 [61.3%], *p* = 0.698) was found. Early postoperative MRI within ≤72 h after resection is obtained by 60.8% of the participants. The most frequent reason for postsurgical imaging was to evaluate the extent of tumor resection (73/120 [60.8%]). In case of residual tumor, 32/120 (26.7%) participants indicated to adjust radiotherapy, 34/120 (28.3%) to consider re-surgery to achieve complete resection and 8/120 (6.7%) to evaluate both.

**Conclusions:**

MRI was the preferred imaging method in the preoperative setting. In the postoperative course, imaging modalities and timing showed high variability. International guidelines for perioperative imaging with special focus on postoperative MRI to assess residual tumor are warranted to optimize standardized management and adjuvant treatment decisions for BM patients.

## Background

Brain metastases (BM) are a major challenge in modern oncology, as the limited treatment options result in high symptomatic burden and poor patient prognosis [1]. Neurosurgical resection represents an important treatment option, especially in patients with solitary BM unknown histology or risk of hydrocephalus [2]. International guidelines from the European Association of Neuro-Oncology (EANO) recommend resection of single, large (diameter ≥ 3 cm) and surgically accessible BM, and for patients presenting severe neurological symptoms and good general health [2]. The neurosurgical goal is to achieve complete resection of BM and subsequent postoperative local radiotherapy/stereotactic radiosurgery (SRS) is able to minimize local tumor recurrence risk [2–4]. However, complete neurosurgical resection might be challenging in some cases as not all BM present with a clear cut, well-demarcated border to the surrounding brain parenchyma [5, 6]. BM lacking a clear-cut demarcation to the surrounding brain parenchyma are at particular risk of incomplete resection, potentially contributing significantly to the local recurrence rate of up to 30.9% after neurosurgical resection [7].

Perioperative imaging is routinely applied to improve neurosurgical resection in glioma patients. Preoperative imaging is used to plan and guide surgery to ensure maximal possible extent of resection and early (< 72 h after resection) postoperative imaging is utilized to identify residual tumor [8–11]. Improved extent of tumor resection has been associated with a longer progression-free survival and overall survival in glioma patients, underscoring the need for optimal tumor resection and the need to address residual tumor formations [11–15].

Computed tomography (CT) scans were shown to be insufficient to differentiate between residual tumor and postoperative bleeding in primary brain tumors, emphasizing the need for postsurgical magnetic resonance imaging (MRI) to guide further treatment options [8, 16]. In order to harmonize the perioperative imaging and optimally guide the therapy approaches, several international guidelines on glioma treatment include detailed imaging recommendations [8, 16]. Currently, postoperative MRI within 72 h is routinely performed at most centers worldwide to investigate the extent of resection after surgery of diffuse infiltrating gliomas [17]. Indeed, postoperative MRI frequently impacts adjuvant treatments as re-resection or adaption of the postoperative treatment can be considered in case of residual tumor [8, 9, 18].

In contrast, perioperative imaging is not standardized in BM patients as so far, no guidelines advocate optimal imaging procedures. Therefore, we aimed to perform a survey analyzing the routine practice of perioperative imaging in patients with BM among the EANO network, to gain insight on the current common practice and especially the variability throughout centers with academic and non-academic backgrounds as well as high and low patient volume centers.

## Methods

### Study design and targeted population

A survey addressing the perioperative management of surgically treated BM patients was designed by the EANO Youngsters committee using an online tool (Survey Monkey© Inc., San Mateo, California, USA, www.surveymonkey.com). The EANO Board members reviewed and approved the survey focus and content. The survey was sent electronically between May and July 2017 to all members of the EANO, and thereby including physicians with a particular focus on neuro-oncology.

### Survey content

This anonymous survey included 19 questions (10 single and 9 multiple-choice questions) addressing the following topics: general information, perioperative standards, preoperative imaging, intraoperative imaging, applied imaging techniques including MRI, CT and positron emission tomography (PET), postoperative imaging and implementation of a dedicated neuro-oncology tumor board (see supplemental material for the full survey questionnaire). Completion of the entire questionnaire took around 5–10 min.

### Statistical analysis

The aim of the current study was to provide a descriptive overview on the current habits and their variability on perioperative imaging within the EANO network. For statistical purposes countries with 3 or less participants were combined in the category ‘others’. High volume centers were defined by a caseload > 50 treated BM patients per year and low volume centers by a caseload ≤50 BM patients per year. Community hospitals, private hospitals and private practices were combined in the category ‘non-academic center’ while university hospitals were referred to as ‘academic center’. Chi square test was used for dichotomous variables. A two-sided *p*-value < 0.05 was considered as significant. All analyses were performed using the software SPSS (IBM SPSS Statistics, Version 25.0. Armonk, NY: IBM Corp.).

## Results

### Physicians’ demographical data

**Fig. 1:**
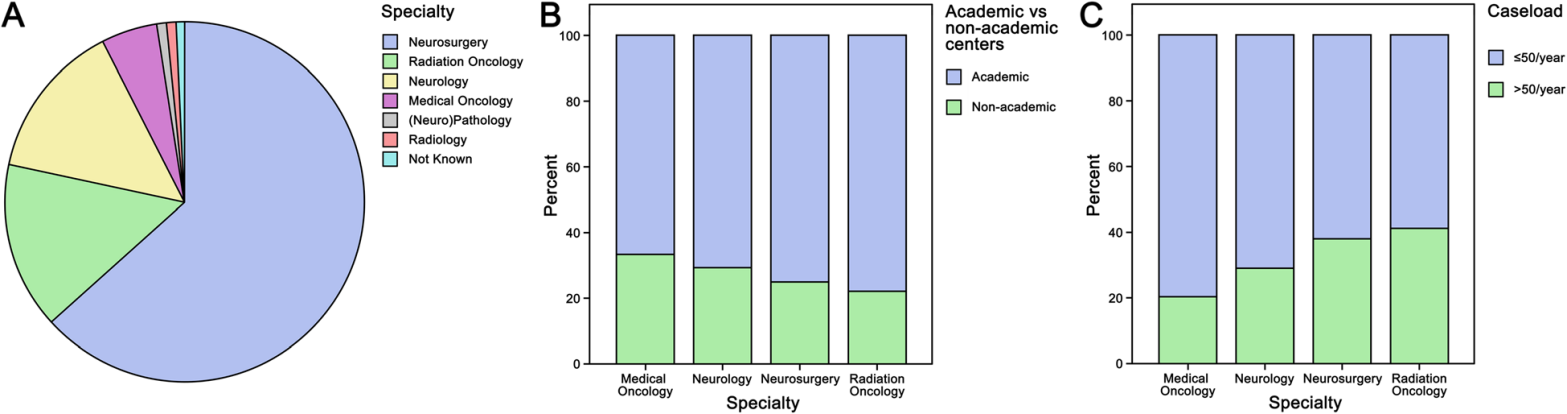
**a** The distribution of the participants throughout the specialties showed the highest participation of neurosurgeons followed by radiation oncologists and neurologists with a similar distribution in **b** academic versus non-academic centers and **c** high versus low volume centers

The survey was distributed via the EANO newsletter to 1054 E-mailing addresses. A total of 120 questionnaires from individual physicians were submitted, resulting in a response rate of 11.4%. The majority of participants were neurosurgeons (76/120 [63.3%]), followed by radiation oncologists (18/120 [15%]), neurologists (17/120 [14.2%]) and medical oncologists (6/120 [5%]; see Table 1 and Fig. 1a for details). Among the participating physicians, 93/120 (77.5%) were from European countries and 27/120 (22.5%) from non-European countries. The majority of participants (89/120 [74.2%]) were located in academic centers, while 31/120 (25.8%) were located in non-academic centers (Fig. 1b). 40/120 (33.3%) physicians worked at high patient volume centers (> 50 BM patient cases per year) and 71/120 (59.2%) in low patient volume centers (≤50 BM patient cases per year). Areas of specialization were evenly distributed within academic center type (see Fig. 1b and supplementary Table 1 for details). Further, no difference regarding specialties according to patient volume center or center localization was observed (see Fig. 1c and supplementary Tables 2 and 3 for details). However, participants from academic centers indicated more frequently to treat a high patient volume compared to participants from non-academic centers (39/40 [97.5%] vs. 1/40 [2.5%], *p* < 0.001).

**Table 1:** Physicians’ demographical data

	n	%
**Specialty**		
Neurosurgery	76	63.3
Radiation Oncology	18	15.0
Neurology	17	14.2
Medical Oncology	6	5.1
(Neuro)Pathology	1	0.8
Radiology	1	0.8
Not Known	1	0.8
**Country**		
Germany	15	12.5
Netherlands	11	9.2
United Kingdom	10	8.3
Switzerland	8	6.7
Italy	7	5.8
Belgium	5	4.2
Austria	4	3.3
Brazil	4	3.3
France	4	3.3
Poland	4	3.3
Spain	4	3.3
United States of America	4	3.3
Others	40	33.3
**Type of institution**		
Academic/University hospital	89	74.2
Community hospital	15	12.5
Private hospital	14	11.7
Private practice	2	1.6
**Number of cases**		
Low volume center (≤50 cases per year)	71	59.2
High volume center (> 50 cases per year)	40	33.3
None	4	3.3
Not known	5	4.2

### Preoperative imaging in patients planned for neurosurgical resection of BM

**Fig. 2:**
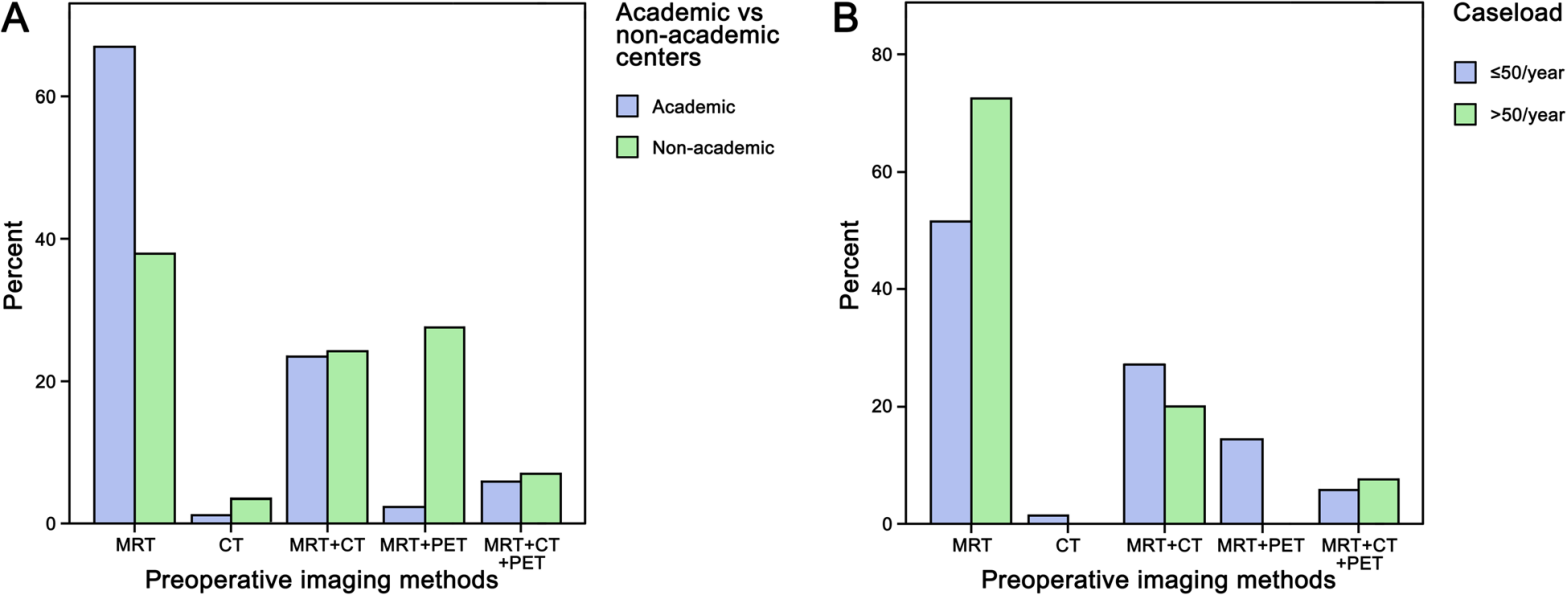
Application of preoperative imaging methods revealed MRI as the most frequently applied preoperative method throughout (**a**) academic versus non-academic and (**b**) low versus high volume centers

Preoperative imaging was routinely performed by 114/120 (95.0%) participating physicians and MRI was the most commonly applied preoperative imaging technique (112/120 [93.3%], Table 2 and Fig. 2a and b). The use of routine preoperative imaging was comparable between academic and non-academic centers (84/89 [94.4%] vs. 28/31 [90.3%]; *p* = 0.435, Fig. 2a), low- and high-patient volume centers (69/71 [97.2%] vs. 40/40 [100%]; *p* = 0.284, Fig. 2b) and European and non-European countries (88/93 [94.6%] vs. 24/27 [88.9%]; *p* = 0.293). Obtaining preoperative imaging was reported at comparable rates for neurosurgeons and participants with other specialty (73/76 [96.1%] vs. 39/44 [88.6%]; *p* = 0.117). Combined preoperative imaging techniques using MRI, CT and/or PET were applied by 44/120 (36.6%) physicians. The combination of MRI with CT was used more often compared to MRI and PET combination (27/120 [22.5%] vs. 10/120 [8.3%]) or the triple combination of MRI, CT and PET (7/120 [5.8%]).

**Table 2:** Pre- and intraoperative imaging of patients treated with resection of BM

	n	%
**Standards for perioperative imaging**		
Yes	94	78.3
No	14	11.7
Not known	12	10.0
**Imaging is supervised by …**		
Neuroradiologist	98	81.7
General radiologist	12	10.0
Neurosurgeon	1	0.8
Not known	9	7.5
**Type of preoperative imaging**		
MRI	112	93.3
CT	36	30.0
PET	17	14.2
**Multimodal preoperative imaging**		
MRI alone	68	56.7
MRI + CT	27	22.5
MRI + PET	10	8.3
MRI + CT + PET	7	5.8
CT alone	2	1.7
Not known	6	5.0
**Preoperative MRI protocol**		
Standard MRI protocol	68	56.7
Advanced imaging protocol	40	33.3
Shortened MRI protocol	2	1.7
Not known	10	8.3
**Intraoperative techniques**		
Neuronavigation	90	75.0
Electrophysiological monitoring/stimulation	56	46.7
Awake surgery	42	35.0
Intraoperative ultrasound	39	32.5
Fluorescence-guided surgery	23	19.2
Intraoperative MRI	9	7.5
Intraoperative CT	3	2.5
Not known	11	9.2

*CT* computed tomography, *MRI* magnetic resonance imaging, *PET* positron emission tomography

### Intraoperative imaging and techniques to guide BM resection

A total of 59/120 (49.1%) physicians reported that intraoperative imaging during neurosurgical resection was conducted at their particular center. The most widely applied intraoperative imaging technique was intraoperative ultrasound (39/120 [32.5%]) followed by intraoperative MRI or CT (12/120 [10.0%]). Availability rate of intraoperative MRI or CT was comparable between academic and non-academic centers (9/12 [75.0%] vs. 3/12 [25.0%]; *p* = 0.945) or high and low patient volume centers (7/11 [63.6%] vs. 4/11 [36.4%]; *p* = 0.981).

Intraoperative neuronavigation was the most frequently applied intraoperative technique for guidance of BM resection (90/120 [75.0%]), followed by electrophysiological monitoring/stimulation (56/120 [46.7%]), and awake surgery (42/120 [35.0%]). 23/120 [19.2%]) physicians indicated to use fluorescence-guided surgery with 5-aminolevulinic acid (5-ALA). The rate of fluorescence-guided surgery in non-academic centers was numerically higher (8/31 [25.8%]) compared to academic centers (15/89 [16.9%]; *p* = 0.202; see Table 2).

### Postoperative imaging after neurosurgical BM resection

**Fig. 3:**
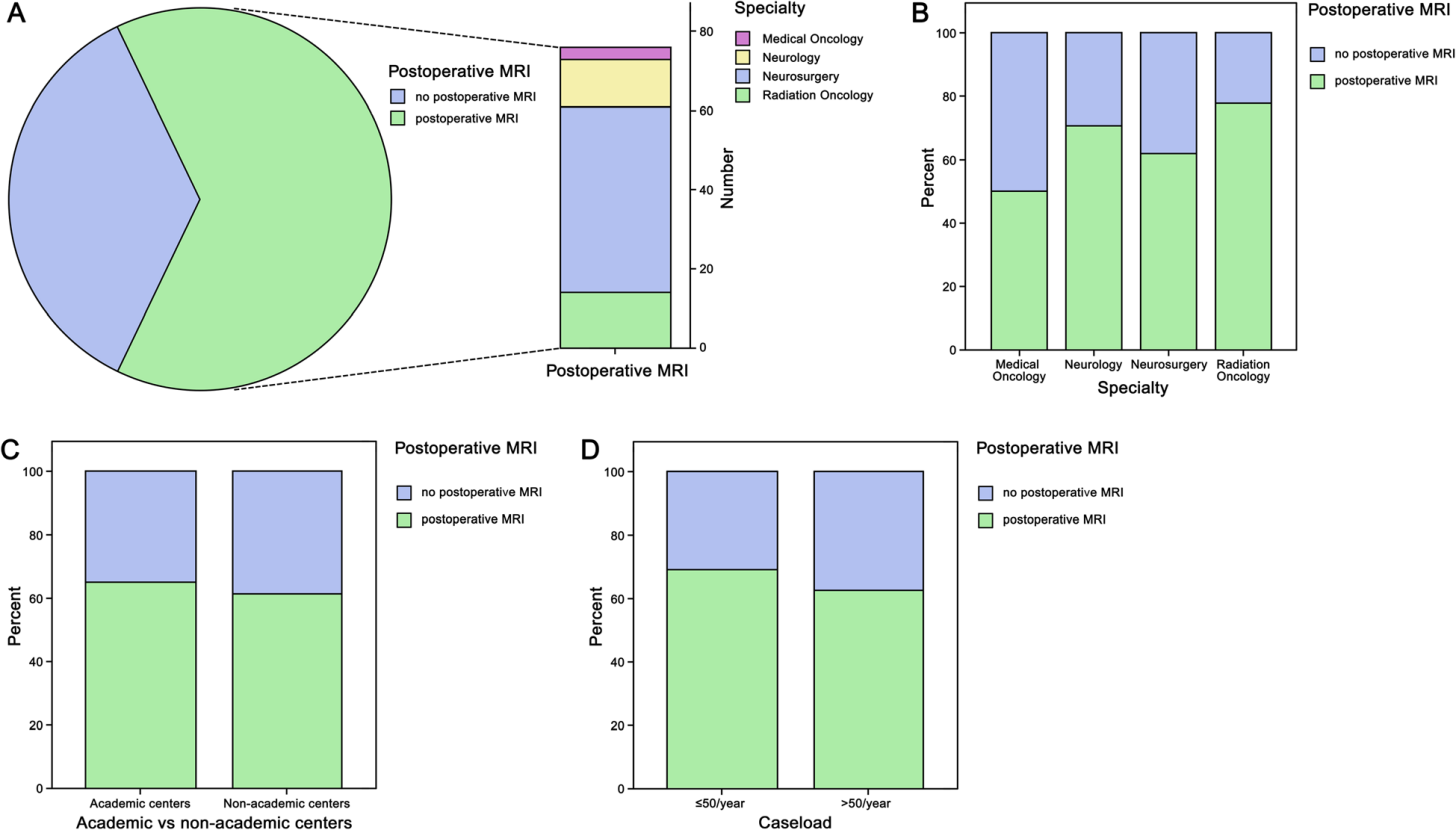
**a**, **b** The application of postoperative MRI was more important for neurosurgeons followed by radiation oncologist and neurologists compared to medical oncologists. **c** Academic versus non-academic as well as **d** low and high volume centers equally performed MRI in the postoperative setting

A total of 106/120 (88.3%) physicians reported to routinely perform postoperative imaging including MRI and/or CT within the first days after neurosurgical resection. The remaining 6 participants stated to perform no postoperative imaging (5/120 [4.2%]) or were not aware of the routine practice at their center (1/120 [0.8%]). 62/120 (51.7%) participants indicated to perform postoperative MRI alone, 29/120 (24.2%) to perform postoperative CT and the residual 15/120 (12.5%) participants stated to prefer the combination of MRI and CT imaging (Fig. 3a and Table 3). Postoperative CT was performed to excluded postoperative complications such as hematoma or ischemia according to 29/120 (24.2%) participants. 10/120 (8.3%) physicians indicated to perform a CT in the postoperative course to evaluate the extent of tumor resection. Medical oncologists (3/6 [50%]) reported the need for a postoperative MRI less frequently compared to neurologists (12/17 [70.6%]), radiation oncologists (14/18 [77.8%]) and neurosurgeons (47/76 [61.8%], *p* = 0.484; Fig. 3a and b). Indication for postoperative MRI was given at comparable rates between participants from academic and non-academic centers (58/89 [65.2%] vs. 19/31 [61.3%], *p* = 0.698; Fig. 3c) as well as from high and low patient volume centers (49/71 [69.0%] vs 25/40 [62.5%], *p* = 0.485; Fig. 3d). Participants from European countries indicated the use of postoperative MRI more frequently compared to participants from non-European countries (64/93 [68.8%] vs. 13/27 [48.1%], *p* = 0.049).

**Table 3:** Postoperative imaging of patients treated with resection of BM

	n	%
**Postoperative imaging**		
Postoperative MRI	77	64.2
Postoperative CT	44	36.7
No postoperative imaging	5	4.2
Not known	1	0.8
**Time point of postoperative MRI**		
≤ 72 h after resection	73	60.8
> 72 h to 7 days after resection	2	1.7
> 7 days to 4 weeks after resection	7	5.8
> 4 weeks to 3 months after resection	18	15.0
> 3 months after resection	4	3.3
Very variable	1	0.8
Not known	15	12.6
**Reasons for postoperative MRI**		
To evaluate the extent of resection	73	60.8
To exclude postoperative complications (hematoma, ischemia ...)	34	28.3
For research purpose	8	6.7
**Parameters influencing time point of postoperative MRI**		
Number of BM	26	21.7
Histology of primary tumor	18	15.0
Previous therapy of BM	18	15.0
GPA class/life expectancy of patient	12	10.0
None	58	48.3
Not known	4	3.3
**Consequences in case of residual tumor**		
Adjustment of the radiotherapy plan	32	26.7
Considering re-do surgery to achieve complete resection	34	28.3
both	8	6.7
**Causes of lack of postoperative MRI**		
Considered unnecessary	17	14.2
No capacity/availability	13	10.8
Due to high costs	9	7.5
Intraoperative MRI already performed	0	0

*BM* brain metastases, *CT* computed tomography, *MRI* magnetic resonance imaging

Early postoperative MRI within ≤72 h after resection was indicated to be routinely performed by 73/120 (60.8%) physicians. The number of BM (26/120 [21.7%]), histology of primary tumor (18/120 [15%]), previous therapies (18/120 [15%]) and the graded prognostic assessment class/life expectancy of patient (12/120 ([10%]) were nominated parameters influencing the time point of postoperative MRI. Evaluating the extent of resection was the most commonly reported reason to perform a postoperative MRI (73/120 [60.8%]). In case of residual tumor in the postoperative MRI, 32/120 (26.7%) participants indicated to adjust the radiotherapy plan, 34/120 (28.3%) to consider re-resection in order to achieve complete and 8/120 (6.7%) stated to consider both.

No availability of postoperative MRI (13/120 [10.8%]) or high costs (9/120 [7.5%]) were the most frequent reasons to omit postoperative MRI.

### Standard operating procedures for perioperative imaging

Local standard operating procedures (SOP) on the perioperative imaging in BM patients were available for 94/120 (78.3%) physicians (Table 2). No difference in the use of local SOP for perioperative imaging between participants from academic and non-academic centers (68/89 [76.4%] vs. 26/31 [83.9%]; *p* = 0.385), high and low patient volume centers (56/71 [78.9%] vs. 35/40 [87.5%]; *p* = 0.256) or European and non-European countries (73/93 [78.5%] vs. 21/27 [77.8%]; *p* = 0.937) was evident.

### Availability of a dedicated neuro-oncology tumor board for BM patients

Treatment plans for BM patients were discussed in a dedicated neuro-oncology tumor board by 98/120 (81.7%) participating physicians. Dedicated neuro-oncology tumor boards were established at comparable rates in academic and non-academic centers (73/89 [82.0%] vs. 25/31 [80.6%]; *p* = 0.864), in high and low patient volume centers (62/71 [87.3%] vs. 34/40 [85%]; *p* = 0.731) and in European vs. non-European countries (77/93 [82.8%] vs. 21/27 [77.8%]; *p* = 0.553). Both pre- as well as additional postoperative discussion of the individual cases were performed by 63/98 (64.2%) physicians.

## Discussion

Neurosurgical resection is an important treatment option in the multimodal management of BM patients [2]. Although BM represent the most common brain tumors, perioperative imaging guidelines for surgically treated BM to standardize optimal adjuvant treatment are so far lacking. The present survey conducted by the EANO Youngsters Committee is the first to evaluate the current perioperative imaging modalities in BM patients. A total of 120 physicians worldwide, from academic as well as non-academic centers, high and low volume centers, European and non-European countries, participated in this survey. The survey revealed that MRI is the preferred perioperative imaging technique and is routinely applied in the preoperative setting, whereas a high variability of postoperative neuroimaging routines (including CT and MRI) was observed throughout the EANO network.

MRI was the most commonly applied preoperative imaging technique, regardless of the investigated center and geographical localization. Preoperative MRI is a broadly established diagnostic tool to plan treatment options of BM including surgery, radiation therapy, radiosurgery and systemic treatments [2, 16, 19–23]. Differentiation of BM from other tumor entities, such as malignant gliomas or lymphomas, as well as pseudoprogression/radionecrosis, is predominately based on preoperative MRI [16, 20, 21, 23]. Aside from diagnostic evaluation of presurgical MRI, this important tool also supports the neurosurgeon’s approach to surgical planning [24–26]. Based on the experiences and recommendations for primary brain tumors, additional diffusion tensor imaging (DTI) can be applied in case of eloquent localizations also in BM patients in order to improve preoperative definition of the surgical strategy as well as subsequent intraoperative navigation to avoid injury of functional white matter tracts [26, 27]. Nevertheless, the so far existing preoperative imaging recommendations from primary brain tumors would need validation in BM patients [28].

Neuronavigation was the most frequently applied intraoperative technique during BM resection, as it represents currently the standard for preoperative planning and intraoperative guidance [29–31]. Furthermore, electrophysiological monitoring/stimulation and awake surgery were used by some of the participating physicians. These techniques are useful to minimize the risk of a new postoperative neurological deficit and thus support the neurosurgeon to achieve safe resection of BM also in eloquent tumor localizations [32–34]. Moreover, one fourth of physicians reported to use fluorescence-guided surgery with 5-aminolevulinic-acid (5-ALA). To date, fluorescence-guided surgery is mainly used for resection of high-grade gliomas, but recently was also described to be useful for intraoperative visualization of BM tissue [7, 35–37]. Intraoperative MRI or CT were infrequently applied, potentially as a consequence of the high costs and the low acceptance in BM surgery. However, due to the frequent lack of clear delineation of BM towards the surrounding brain parenchyma intraoperative techniques and especially 5-ALA might be of additional value to ensure optimal extent of resection [6].

The majority of physicians performed a postsurgical MRI, although only approximately half of the participating physicians indicated to perform early postoperative MRI within 72 h after tumor resection. No differences in the use of postsurgical MRI were evident between academic and non-academic centers, while European participants reported the use more frequently than non-European participants. Interestingly, differences were observed according to the medical specialties. Oncologists reported less frequent use of post-surgical imaging compared to the other specialties. EANO guidelines on diagnosis and treatment of BM recommend postoperative MRI to guide adjuvant radiotherapy applied to the resection cavity as the postsurgical resection cavity volume is smaller than preoperative BM volume [2]. However, no recommendation on the optimal timepoint for postoperative MRI after BM resection is given in the current version. As indeed timing is stated to be not relevant for this particular postoperative application [2]. Importantly, postsurgical changes, such as ischemia, bleeding, or postsurgical gliosis frequently occur and may mimic a residual tumor in case of MRI is performed later than 72 h after resection [8]. In glioma surgery, several guidelines stress the importance of an early postoperative MRI within 72 h after surgery to reliably differentiate postsurgical changes and residual tumor and guide the subsequent therapeutic approach [8]. A recent publication revealed residual tumor on early postoperative MRI in 20% of BM cases, although 92.3% of these were classified as complete resection by the surgeon [38]. These observations further stress the importance of accurately accessing the tumor residue with early postsurgical MRI and including this information in the further treatment plan.

More than half of the participants indicated to adjust the radiotherapy plan or even consider re-do surgery to achieve complete resection in case of residual tumor in the early postoperative MRI. Indeed, adjuvant therapy after BM resection has been controversially discussed. Whole brain radiotherapy (WBRT) has been shown to increase local tumor control as well as the distant brain control [4, 39, 40]. However, WBRT had no impact on overall survival [4, 39, 40]. Due to potential neuro-cognitive decline, WBRT is currently controversial in EANO guidelines [41, 42]. Adjuvant Stereotactic fractionated radiotherapy (SFRT) or stereotactic radiosurgery (SRS) of the resection cavity has been suggested to increase the local disease control [33, 43]. So far only very small studies address the clinical impact of early postsurgical imaging in BM [38, 44]. One recent publication stressed that routine postoperative MRI is unnecessary because patients with small residual tumor did not undergo any changes of treatment plan [44]. In this retrospective study, the authors recommended postoperative imaging only in case of neurological deficits, concerns about large amounts of residual tumor or intraoperative complications [44]. However, considering the new opportunities of adjuvant SRS/SFRT, this might not hold true in modern BM management and should be investigated in further clinical trials.

The majority of participants of our survey stated to conduct perioperative imaging in BM according to local SOP. These findings were independent of academic vs. non-academic centers or European vs. non-European countries. Guidelines on the perioperative imaging are well established in primary brain tumors, but are missing so far for BM [8]. Especially in high-grade glioma patients, the evaluation of the extent of resection plays an important role for prognosis [13, 45]. Several studies indicated a better progression-free and overall survival in case of complete resection of the contrast enhancing tumor [13, 45].

Based on the results of our survey, international guidelines for perioperative imaging in BM are warranted to ensure a standardized optimal postoperative treatment approach and to provide a comparable standard through centers. In our view, the most appropriate method of perioperative imaging in BM represents MRI. In this sense, we recommend performing a standardized preoperative MRI protocol for optimal tumor diagnosis, selection of the appropriate treatment option and preoperative planning. After surgery of BM, we suggest conducting a standardized early postsurgical MRI within 72 h after surgery to evaluate especially the extent of tumor resection and thus optimize subsequent treatment allocation. In case of a significant postsurgical residual tumor, we propose to consider a re-do surgery or adjustment of the radiotherapy plan.

Our survey was performed anonymously to reduce a potential bias based on reporting the treatment institution. However, in consequence we did not include the identification of the center and therefore cannot address how many participants from the same center answered the survey. Certainly, physicians with a particular focus on BM treatment were more likely to answer the survey out of interest and therefore bias the given results. Nevertheless, we provide the first investigation of the current practice of perioperative imaging in BM patients, showing a particular variability in the postoperative imaging modalities and therefore stressing the need for international guidelines to harmonize optimized perioperative treatment algorithms.

## Conclusion

In conclusion, we were able to conduct the first international survey on perioperative imaging in BM patients. Although the majority of included physicians routinely use perioperative MRI, only half obtain early postoperative MRI to reliably identify residual tumor. No availability of postoperative MRI or high costs were the most frequent reasons to omit postoperative MRI. International guidelines on the perioperative imaging may help to optimize treatment approaches and ensure a high level of standard treatment throughout centers.

## Supplementary information

**Additional file 1:** Survey of the EANO Youngster - "Evaluation of perioperative management of surgically treated brain metastases".

**Additional file 2: Supplementary Table 1.** Specialization distribution within academic centers and non-academic centers. **Supplementary Table 2.** Specialization distribution within European and non-European-countries. **Supplementary Table 3.** Specialization distribution within high-volume and low-volume centers.

## Data Availability

The datasets used and/or analyzed during the current study are available from the corresponding author on request.

## Abbreviations

5-ALA: 5-aminolevulinic acid
BM: Brain metastases
CT: Computed tomography
DTI: Diffusion tensor imaging
EANO: European Association of Neuro-Oncology
MRI: Magnetic resonance imaging
PET: Positron emission tomography
SFRT: Stereotactic fractionated radiotherapy
SOP: Standard operating procedures
SRS: Stereotactic radiosurgery
WBRT: Whole brain radiotherapy
