# Supplementary material for: Perioperative imaging in patients treated with resection of brain metastases: a survey by the European Association of Neuro-Oncology (EANO) Youngsters committee
Source: BMC Cancer. 2020 May 12;20:410. doi: 10.1186/s12885-020-06897-z (PMC7216695; doi:10.1186/s12885-020-06897-z)
Supplement: Supplementary file 1 — Additional file 1: Survey of the EANO Youngster - "Evaluation of perioperative management of surgically treated brain metastases". [file 12885_2020_6897_MOESM1_ESM.pdf]

# EVALUATION OF PERIOPERATIVE MANAGEMENT OF SURGICALLY TREATED BRAIN METASTASES

## Survey of the EANO Youngsters

## Aim of our survey

A previous national survey revealed differences in the perioperative management of patients with brain metastases. Consequently, we designed this survey to analyze the different international perioperative management strategies of surgically treated brain metastases.

Thank you very much for your participation!

On behalf of the EANO Youngsters Group

Barbara Kiesel, MD
Anna Berghoff, MD
Carina Thomé, M.Sc.

MEDIZINISCHE UNIVERSITÄT WIEN

## General information

### 1. What is your field of expertise?

- ◯ Medical Oncology
- ◯ Neurology
- ◯ Neurosurgery
- ◯ (Neuro)Pathology
- ◯ Radiation Oncology
- ◯ Radiology
- ◯ Other (please specify)

### 2. In which country are you working?

### 3. What type of institution do you work at?

- ◯ Academic/University hospital
- ◯ Community hospital
- ◯ Private hospital
- ◯ Private practice

## Number of resected brain metastases

### 4. How many resections of brain metastases are approximately performed at your institution each year?

- ◯ < 20 cases per year
- ◯ 20 - 50 cases per year
- ◯ > 50 - 100 cases per year
- ◯ > 100 cases per year
- ◯ None
- ◯ Not known

## Standards for perioperative imaging

### 5. Do you use standard operating procedures for perioperative imaging in brain metastases at your institution?

- Yes
- No
- Not known

### 6. Perioperative imaging (MRI, CT) of brain metastases at your institution is supervised by a ...

- Neuroradiologist
- General Radiologist
- Not known
- Other (please specify)

## Preoperative imaging

### 7. What type(s) of neuroradiological imaging is/are usually performed for preoperative planning/diagnosis before resection of brain metastases at your institution?

(more than one answer possible)

- [ ] Magnetic resonance imaging (MRI)
- [ ] Computertomography (CT)
- [ ] Positron emission tomography (PET)
- [ ] Not known
- [ ] Other (please specify)

## Type of preoperative MRI

### 8. What MRI sequences are usually performed for preoperative evaluation of brain metastases at your institution?

- ◯ Shortened MRI protocol consisting mainly of T1 weighted images with/without contrast media
- ◯ Standard MRI protocol (FLAIR, T2, T1 weighted images with/without contrast media)
- ◯ Advanced imaging protocol including for example MR-spectroscopy or perfusion MRI
- ◯ Not known
- ◯ Other (please specify)

## Intraoperative techniques

### 9. Which intraoperative technique(s) is/are usually applied during resection of brain metastases at your institution?

(more than one answer possible)

- [ ] Neuronavigation
- [ ] Intraoperative CT
- [ ] Intraoperative MRI
- [ ] Intraoperative ultrasound
- [ ] Fluorescence-guided surgery
- [ ] Electrophysiological monitoring/stimulation
- [ ] Awake surgery
- [ ] Not known
- [ ] Other (please specify)

## Type of postoperative imaging

### 10. Which type(s) of postoperative imaging is/are routinely performed in the first days after complication-free resection of brain metastases at your institution?

(more than one answer possible)

- [ ] Magnetic resonance imaging (MRI)
- [ ] Computertomography (CT)
- [ ] No imaging
- [ ] Not known

## Aim of postoperative MRI

### 11. Why is routine postoperative MRI performed after complication-free resection of brain metastases at your institution?

(more than one answer possible)

- [ ] To exclude postoperative complications such as hematoma or ischemia
- [ ] To evaluate the extent of resection
- [ ] For research purpose
- [ ] Not known
- [ ] Other (please specify)

### 12. Postoperative MRI reveals a significant unexpected residual tumor after resection of a brain metastasis. What are the consequences at your institution?

- ○ Considering re-do surgery to achieve complete resection
- ○ Adjustment of the radiotherapy plan
- ○ None
- ○ Not known
- ○ Other (please specify)

## Aim of postoperative CT

### 13. Why is routine postoperative CT performed after complication-free resection of brain metastases at your institution?

(more than one answer possible)

- [ ] To exclude postoperative complications such as hematoma or ischemia
- [ ] To evaluate the extent of resection
- [ ] For research purpose
- [ ] Not known
- [ ] Other (please specify)

## Causes for lack of implementation of postoperative MRI

### 14. Why is a postoperative MRI after complication-free resection of brain metastases not routinely performed at your institution?

(more than one answer possible)

- ☐ Due to high costs
- ☐ No capacity/availability
- ☐ Intraoperative MRI already performed
- ☐ Considered unnecessary
- ☐ Not known
- ☐ Other (please specify)

## Causes for lack of implementation of postoperative MRI

### 15. Why is a postoperative MRI after complication-free resection of brain metastases not routinely performed at your institution?

(more than one answer possible)

- ☐ Due to high costs
- ☐ No capacity/availability
- ☐ Intraoperative MRI already performed
- ☐ Considered unnecessary
- ☐ Not known
- ☐ Other (please specify)

EVALUATION OF PERIOPERATIVE MANAGEMENT OF SURGICALLY TREATED BRAIN METASTASES

Survey of the EANO Youngsters

## Timing of postoperative MRI

### 16. In which time interval is the first postoperative MRI routinely performed after complication-free resection of brain metastases at you institution?

- ≤ 72 hours after resection
- > 72 hours to 7 days after resection
- > 7 days to 4 weeks after resection
- > 4 weeks to 3 months after resection
- > 3 months after resection
- Not known
- Other (please specify)

### 17. Which of the following parameters influences the timepoint of the first postoperative MRI after complication-free resection of brain metastases at your institution?

(more than one answer possible)

- Number of brain metastases (singular vs. multiple)
- Histology of the primary tumor
- Previous therapy for brain metastases
- GPA class/life expectancy of the patient
- None of the above
- Not known
- Other (please specify)

## Tumor Board

### 18. Are patients with surgically treated brain metastases routinely discussed in an interdisciplinary Tumor Board at your institution?

(more than one answer possible)

- ☐ Yes, preoperative discussion
- ☐ Yes, postoperative discussion
- ☐ No discussion
- ☐ Not known

### 19. Does a (neuro)radiologist attend the interdisciplinary Tumor Board and discuss neuroimaging with clinicians?

- ◯ Yes, always
- ◯ Yes, occasionally
- ◯ Only on request
- ◯ No
- ◯ Not known

### 20. Do you have any other comments, questions, or concerns?

Thank you very much!
