# Supplementary material for: Perioperative imaging in patients treated with resection of brain metastases: a survey by the European Association of Neuro-Oncology (EANO) Youngsters committee
Source: BMC Cancer. 2020 May 12;20:410. doi: 10.1186/s12885-020-06897-z (PMC7216695; doi:10.1186/s12885-020-06897-z)
Supplement: Supplementary file 2 — Additional file 2: Supplementary Table 1. Specialization distribution within academic centers and non-academic centers. Supplementary Table 2. Specialization distribution within European and non-European-countries. Supplementary Table 3. Specialization distribution within high-volume and low-volume centers. [file 12885_2020_6897_MOESM2_ESM.docx]

**SUPPLEMENTARY TABLES**

**Supplementary table 1: Specialization distribution within academic centers and non-academic centers**

| **Specialty** | **Academic center** | **Non-academic center** |
| --- | --- | --- |
|  |  |  |
| Neurosurgery | 57 (64.0%) | 19 (61.3%) |
| Radiation Oncology | 14 (15.7%) | 4 (12.9%) |
| Neurology | 12 (13.5%) | 5 (16.1%) |
| Medical Oncology | 4 (4.6%) | 2 (6.5%) |
| (Neuro)Pathology | 1 (1.1%) | 0 |
| Radiology | 1 (1.1%) | 0 |
| Not Known | 0 | 1 (3.2%) |
|  |  |  |

**Supplementary table 2: Specialization distribution within European and non-European-countries**

| **Specialty** | **European countries** | **Non-European countries** |
| --- | --- | --- |
|  |  |  |
| Neurosurgery | 61 (65.6%) | 15 (55.6%) |
| Radiation Oncology | 14 (15.1%) | 4 (14.8%) |
| Neurology | 12 (12.9%) | 5 (18.5%) |
| Medical Oncology | 4 (4.2%) | 2 (7.4%) |
| (Neuro)Pathology | 1 (1.1%) | 0 |
| Radiology | 0 | 1 (3.7%) |
| Not Known | 1 (1.1%) | 0 |
|  |  |  |

**Supplementary table 3: Specialization distribution within high-volume and low-volume centers**

| **Specialty** | **High-volume**  **(>50 BM/year)** | **Low-volume (≤50 BM/year)** |
| --- | --- | --- |
|  |  |  |
| Neurosurgery | 47 (62.7%) | 28 (70.0%) |
| Radiation Oncology | 11 (14.7%) | 7 (17.5%) |
| Neurology | 11 (14.7%) | 4 (10.0%) |
| Medical Oncology | 4 (5.3%) | 1 (2.5%) |
| (Neuro)Pathology | 1 (1.3%) | 0 |
| Not Known | 1 (1.3%) | 0 |
|  |  |  |
| BM…brain metastases | | |
